# Long-segment intramedullary spinal dermoid

**DOI:** 10.4103/0971-3026.50840

**Published:** 2009-05

**Authors:** NC Sharma, Tushar Chandra, Anshu Sharma, Manish Bajaj, Ravinder Kundu

**Affiliations:** Department of Radiodiagnosis, R.N.T. Medical College and Associated Group of Hospitals, Udaipur (Rajasthan) 313 001, India; 1 MR Centre, M.B. Govt. Hospital, Udaipur (Rajasthan) - 313 001, India

**Keywords:** Dermoid, intramedullary, spinal

## Abstract

A 30-year-old man presented with a fairly large intramedullary mass lesion involving virtually the entire spinal cord. It was hyperintense on both T1W and T2W sequences, with signal suppression on fat-saturation images. Subsequent noncontrast CT scan of the spine confirmed the presence of fat and calcification within the lesion, thus leading to the diagnosis of an intramedullary dermoid.

Spinal dermoids are rare, benign, slow-growing tumors arising from more than one of three primitive germ cell layers.[1] They are intradural, and sometimes intramedullary, lesions. Imaging may demonstrate areas of fat and calcification within them.[2] We report a case of a long-segment intramedullary dermoid extending from the cervical region to the conus medullaris.

## Case Report

**Figure 1 (A,B):**
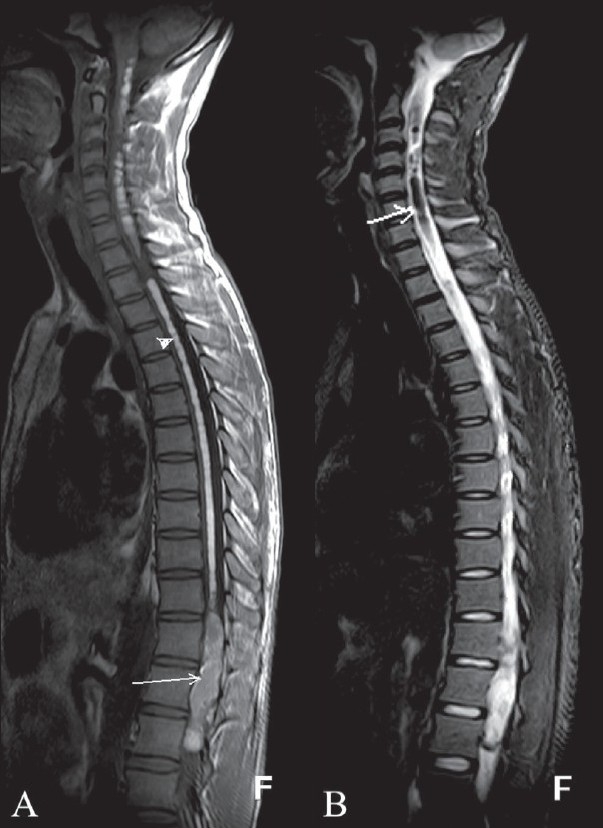
T1W (A) sagittal MRI image shows a heterogeneous intramedullary lesion (arrow) causing expansion of the conus medullaris region with hyperintense intramedullary signal (arrowhead) extending from the cervical to the lumbar regions. The sagittal T2W fat-saturation image (B) shows suppression of signal (arrow) in the upper spinal cord.

**Figure 2 (A–C):**
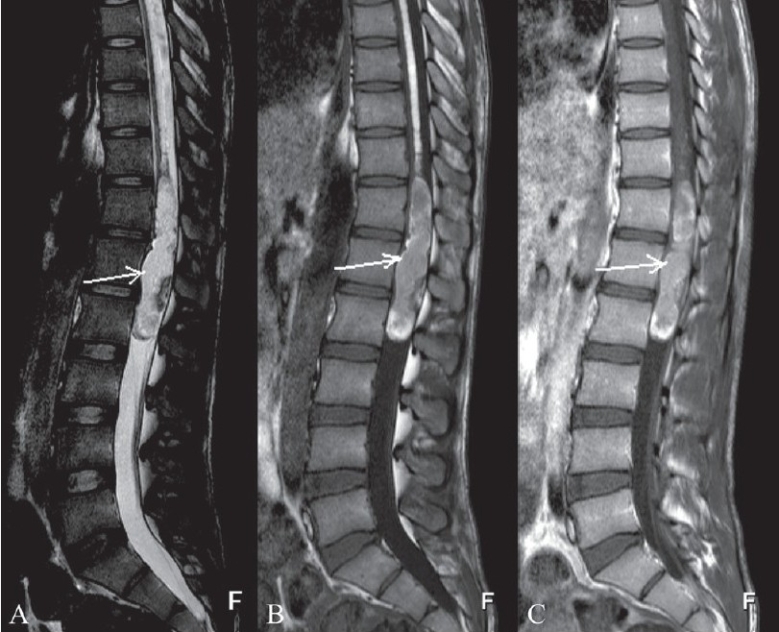
Sagittal T2W (A) and T1W (B) MRI images show a heterogeneous signal intensity mass (arrows) in the conus medullaris region with heterogeneous enhancement in the post-contrast T1W fat-saturation image (C).

**Figure 3 (A–C):**
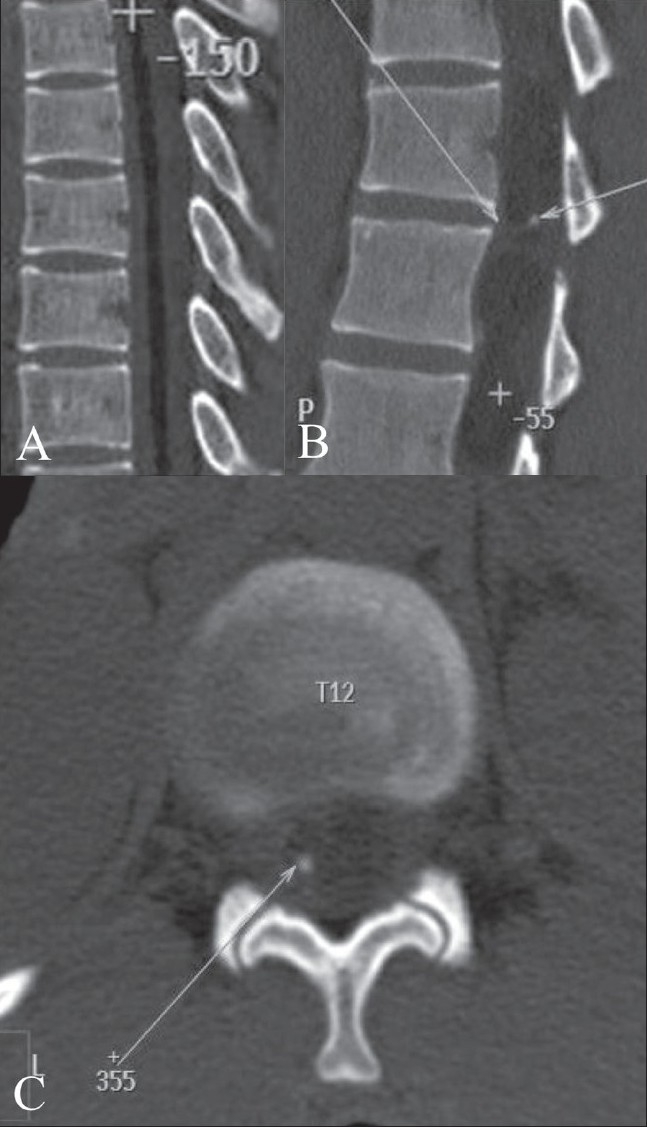
Non-contrast sagittal CT scan reconstructed images show intramedullary fat (+) in the upper spinal cord (A) and a mass in the conus region (long arrow) with areas of fat (+) and calcification (short arrow) (B). Noncontrast axial CT scan (C) shows a focal area of calcification (arrow) within the lesion.

A 30-year-old male presented with pain in the right lower limb for 1 year. He also complained of difficulty in passing urine for 3 months. On examination, saddle anesthesia was noted, with grade 3 power in the left lower limb. MRI of the lumbosacral spine revealed a fairly large intramedullary mass lesion that appeared hyperintense on both T1W and T2W sequences, with signal suppression on fat-saturation images [Figures 1A, B and 2A]. This lesion had three separate segments: one segment extended from the base of the skull to the D1 level, the second from D2 to D11, and the third segment from D11-D12 to L2, in the conus medullaris. The segments in the cervical region and the conus medullaris showed lobulated margins, with mild expansion of the cord and heterogeneous signals. Prominent flow voids were also seen in the cervical region. The segment in the dorsal region of the cord showed well-defined regular outlines and lay centrally in the intramedullary compartment. On the postcontrast study, there was mild heterogeneous enhancement of the mass lesion at the conus medullaris [Figure 2B, C]. These findings were suggestive of a fairly large fat-containing mass lesion, most likely an intramedullary dermoid. Subsequent noncontrast CT spine with sagittal reconstructions confirmed the presence of fat and calcification within the lesion [Figure 3A–C], thus supporting the diagnosis of an intramedullary dermoid. The patient refused surgery and therefore histopathological confirmation could not be obtained.

## Discussion

Dermoid tumors constitute 1.1% of intraspinal tumors.[3] They are thought to arise from the inclusion of ectopic embryonic rests of ectoderm within the spinal canal at the time of neural tube closure during embryonic development.[4] The lumbosacral region is the most common site to be affected (60%).[5]

Although dermoid tumors develop from the embryonic period, they are slow-growing lesions and do not cause symptoms till adult life.[3] Most dermoid tumors become clinically apparent during the second or third decade of life.[6] There is a slight male predominance.

They may be associated with other forms of dysraphism and in about 20% of cases, a dorsal dermal sinus can be seen.[1] Other associations include bony malformations and tethered cord.[7]

The combination of fluid, soft tissue, calcium, and fat is diagnostic of a dermoid tumor. Because of the varying amounts of soft tissue, fat, calcium, and hemorrhage, MRI typically demonstrates heterogeneous signal intensity.[1] The relatively high signal from fat on MRI, especially the bright signal on T1W images, makes identification of the lipid component easy.[7] This can be confirmed by demonstrating low attenuation (negative Hounsfield numbers) on a nonenhanced CT scan, as was done in our case.

Dermoid tumors may have two distinct portions: a lipid part and a more solid or more fluid part. In our case, the lipid component extended from the intramedullary portion of the cervical spinal cord to the thoracolumbar region, with the more solid portion of the tumor being seen in the region of the conus medullaris. The soft tissue component usually enhances after administration of intravenous contrast medium.

To the best of our knowledge, a long-segment intramedullary dermoid is extremely rare. In our patient, the intramedullary dermoid extended from the cervical spinal cord to the conus medullaris; such a case has not been reported before.

In conclusion, MRI is helpful in detecting intraspinal dermoid tumors and delineating their extent. CT plays an important complementary role in identifying areas of fat and calcification.
